# Supplementary material for: Targeting diamondback moths in greenhouses by attracting specific native parasitoids with herbivory-induced plant volatiles
Source: R Soc Open Sci. 2020 Nov 11;7(11):201592. doi: 10.1098/rsos.201592 (PMC7735346; doi:10.1098/rsos.201592)
Supplement: Supplemental materials and figures;Supplemental data (Figure 1);Supplemental data (Figure 2);Supplemental data (Figure 3);Supplemental data (Fig S1 and S2) [file rsos201592supp1.docx]

Supplemental materials, Figure 1 and 2

1. Methods

1.1. Preliminary greenhouse experiments in Miyama greenhouses

We conducted the experiments using dispensers with 4.25mg attractant per dispenser and honey feeders. The setting design of the dispensers and feeders were the same as that in 2006 and 2008. We observed six treated and six untreated greenhouses from 2 May to 21 October 2005. During the experiment, we observed randomly chosen plants in a single observation to count the number of DBMs (adults and larvae): 200 plants of less than 20 cm height, 100 plants of less than 40 cm height, and 50 plants of more than 40 cm height. While systematically walking along all the greenhouse paths (walking observation), we also observed DBM adults that had settled on, or were taking off from the mizuna plants. Additionally, we observed the cocoons of *C. vestalis* in greenhouses. We observed each greenhouse every one or two weeks. The dispensers and feeders were exchanged every observation day. The monthly incidence of DBM occurrences in treated greenhouses and those in the control greenhouses were compared.

1.2. Plants and insects

Komatsuna plants (*Brassica rapa* var. *perviridis*) and mizuna plants (*Brassica rapa* var. *nipposinica*) were used in rearing DBM larvae and experiments. The plants were cultivated in a greenhouse (25 ± 3°C; 60 ± 10% RH; 16 h light:8 h dark photoperiod). One or five plants per plastic pot (upper pot diameter 9 cm; lower pot diameter 7 cm; pot depth 7 cm) were grown from seed for four to five weeks.

DBM larvae were collected in a field near Kyoto, Japan, and mass-reared on potted komatsuna plants in a climate-controlled room (25 ± 3°C; 60 ± 10% RH; 16 h light:8 h dark) to obtain adults. Eggs were collected every day, and hatched larvae were reared on cut plants in small cages (25 × 15 × 10 cm height). Newly emerged adults of DBM were maintained separately in acrylic cages (35 × 25 × 30 cm height) and provided with a 50% (v/v) honey solution as food in a climate-controlled room (25 ± 3°C; 60 ± 10% RH; 16 h light:8 h dark) to ensure mating. After four days, DBM females were used for experiments.

1.3. Effects of the attractant on the performance of DBM

Prior to the field experiments, we studied whether the blend of the four HIPVs affected the interaction between plants and DBM (either adults or larvae). We evaluated the effects of exposure of the attractant to mizuna plants on the pupation rate of DBM larvae and on the weight of DBM pupae on the plants in a climate-controlled room (25 ± 2°C; 60 ± 10% RH; 16 h light:8 h dark).

As mentioned above, dispensers containing 425 mg of the attractant as a 5% solution in TEC were used as the volatile source. One mizuna plant (ca. 15 cm tall) per pot was placed in a plastic box (18 × 32 × 20 cm^3^) with a nylon-gauze-covered window with one dispenser contained the attractant. In the control, the dispenser contained only TEC. A second stadium DBM larva was placed onto each mizuna plant. We checked the pupation of the larvae 4, 6 and 8 days after the initiation of the experiment. Two larvae and one larva were lost in the treated and control experiments, respectively. None of the larvae became pupae at day 4, and all the larvae became pupae at day 8. Thus, we compared the pupation rates at day 6. The weight of pupae was also measured. The experiments were repeated 20 times in four separate experimental days.

We also studied the direct effects of the attractant on oviposition behaviour of DBM females. One mizuna plant per pot was placed in plastic box (18 × 32 × 20 cm^3^) in a climate-controlled room (25 ± 2°C; 60 ± 10%; 16 h light:8 h dark) with either a dispenser containing of the attractant or a dispenser containing only TEC. We released three mated females of DBM into the box at 15:00 and counted the number of eggs laid on each plant 24 hours later. The experiments were repeated 10 times in four separate experimental days.

1.4. Statistics

To analyse the effects of the treatment, month, and their interaction on the DBM occurrence rate, we used a generalized linear mixed model (GLMM) with a binomial distribution and logit-link using the function glmer in the package lme4 version 1.17 [3] in R version 3.3.3 [4]. The greenhouse was a random effect in all models. Significant values from the GLMMs were calculated from type II Wald chi-square tests using the ANOVA function in the ‘car’ package version 2.1.4 [5].

In the experiment on the performance of DBM, the pupation rate of DBM were analysed using Fisher’s exact test in R version 3.3.3 [1]. The pupal weight and the number of eggs of DBM were analysed using *t* test in JMP [2]. All data of the pupal weight were Box-Cox transformed using JMP before the statistical analyses.

2. Results

2.1. Preliminary greenhouse experiments in Miyama greenhouses

In the greenhouse experiments, the monthly incidences of DBM occurrence did not differ significantly between the treated and control greenhouses (Fig. 1). This incidence was significantly affected by month and marginally affected by the interaction (treatment × month) (Fig. 1).

2.2. Effects of the attractant on the performance of DBM

The pupation rates on unexposed plants and the attractant-exposed plants did not differ significantly in either males or females (male: *P* = 0.5658; female: *P* = 1; Supplemental Fig. 2A). The pupal weight was also unaffected by exposure to the attractant ((male: t = 1.3108, df = 18, *P* = 0.2069; female: t = 0.0020, df = 15, *P* = 0.9985; Fig. 2B). We also analysed the direct effects of the attractant on the oviposition behaviour of DBM females, and found that the number of eggs was not affected by the presence or absence of the attractant (*t* = 0.2875, df = 18, *P* = 0.7770; Fig. 2C).

Reference

1. Bates D, Maechler M, Bolker B, Walker S. 2015 Fitting Linear Mixed-Effects Models Using lme4. *J. Stat. Soft. e* :67: 1-48.

2. SAS Institute 2018. JMP ver 14.2.0. SAS Institute, Inc., Cary, NC, USA

3. Bates D, Maechler M, Bolker B, Walker S. 2015 Fitting Linear Mixed-Effects Models Using lme4. J. Stat. Soft. e :67: 1-48.

4. R Core Team. 2017 R: A language and environment for statistical computing. R Foundation for Statistical Computing, Vienna, Austria. URL:

http://www.R-project.org/. Accessed April 6: 2017.

5. Fox J, Weisberg S. 2011 An R Companion to Applied Regression, 2nd edition. (Sage, Thousand Oaks, CA)

.

Supplemental Figure 1. Relative number of greenhouses in which more than one DBM was observed per month (the monthly incidence of DBM occurrence). ND: Moth was not detected.

Supplemental Fig. 2. Effects on DBM of exposure to attractant on mizuna plants. (A) pupation rate; (B) pupal weight (mg); (C) number of DBM eggs oviposited. NS: not significantly different.
